# The Different Faces of Insomnia

**DOI:** 10.3389/fpsyt.2021.683943

**Published:** 2021-06-29

**Authors:** Ingo Fietze, Naima Laharnar, Volker Koellner, Thomas Penzel

**Affiliations:** ^1^Department of Internal Medicine and Dermatology, Interdisciplinary Center of Sleep Medicine, Charité - Universitätsmedizin Berlin, Berlin, Germany; ^2^Department of Behavioral Therapy and Psychosomatic Medicine, Rehabilitation Center Seehof, Federal German Pension Agency, Seehof, Germany; ^3^Department of Biology, Saratov State University, Saratov, Russia

**Keywords:** insomnia, phenotypes, subtypes, heterogeneity, symptom, progression, questionnaire, screening

## Abstract

**Objectives:** The identification of clinically relevant subtypes of insomnia is important. Including a comprehensive literature review, this study also introduces new phenotypical relevant parameters by describing a specific insomnia cohort.

**Methods:** Patients visiting the sleep center and indicating self-reported signs of insomnia were examined by a sleep specialist who confirmed an insomnia diagnosis. A 14-item insomnia questionnaire on symptoms, progression, sleep history and treatment, was part of the clinical routine.

**Results:** A cohort of 456 insomnia patients was described (56% women, mean age 52 ± 16 years). They had suffered from symptoms for about 12 ± 11 years before seeing a sleep specialist. About 40–50% mentioned a trigger (most frequently psychological triggers), a history of being bad sleepers to begin with, a family history of sleep problems, and a negative progression of insomnia. Over one third were not able to fall asleep during the day. SMI (sleep maintenance insomnia) symptoms were most frequent, but only prevalence of EMA (early morning awakening) symptoms significantly increased from 40 to 45% over time. Alternative non-medical treatments were effective in fewer than 10% of cases.

**Conclusion:** Our specific cohort displayed a long history of suffering and the sleep specialist is usually not the first point of contact. We aimed to describe specific characteristics of insomnia with a simple questionnaire, containing questions (e.g., ability to fall asleep during the day, effects of non-medical therapy methods, symptom stability) not yet commonly asked and of unknown clinical relevance as yet. We suggest adding them to anamnesis to help differentiate the severity of insomnia and initiate further research, leading to a better understanding of the severity of insomnia and individualized therapy. This study is part of a specific Research Topic introduced by Frontiers on the heterogeneity of insomnia and its comorbidity and will hopefully inspire more research in this area.

## Introduction

Insomnia is one of the most frequent sleep disorders with continuously increasing prevalence. About 30–50% of the US adult population exhibit insomnia symptoms, 15–20% display a short-term insomnia of <3 months, and 5–15% display a chronic insomnia of >3 months (1–3). Common diagnostic manuals include the ICSD-3 (International Classification of Sleep Disorders, 3^rd^ Edition, American Academy of Sleep Medicine 2014) and the DSM-5 (Diagnostic and Statistical Manual of Mental Disorders, 5^th^ Edition, American Psychiatric Association 2013) (4, 5). Main characteristics of insomnia include dissatisfaction with sleep quantity and quality with one or more of the following symptoms: difficulties initiating sleep, difficulties maintaining sleep (frequent or prolonged awakenings with problems returning to sleep again), and early morning awakening (occurring earlier than desired after a total sleep time of only 3–5 h with the inability to return to sleep). The disturbed sleep is associated with stress, psychological strain and suffering, as well as impairment in social, occupational, and other important areas of functioning. Complaints include fatigue, exhaustion, lack of energy, daytime sleepiness, cognitive impairment (e.g., attention, concentration, and memory), mood swings (e.g., irritability, dysphoria), impaired occupational functioning and impaired social functioning. The symptoms occur for at least 3 nights per week for at least 3 months and occur despite an adequate sleep environment.

Previous dichotomization of insomnia in primary and secondary (or comorbid) insomnia has been abandoned with the new editions of the DSM-5 and ICSD-3. Currently, insomnia is mostly characterized by the common phenotypes of sleep onset insomnia (SOI insomnia, difficulty falling asleep), sleep maintenance insomnia (SMI insomnia, difficulty staying asleep), early morning awakenings insomnia (EMA insomnia), and a combination of those. Another categorization follows the timeframe of being an acute (<1 month), subacute (1–3 months), and chronic insomnia (>3 months) (4, 5). While other sleep disorders (e.g., sleep apnea) are categorized by severity into mild, moderate, or severe, which has important implications for the choice of therapy, insomnia still lacks such a classification. The Insomnia Severity Index (ISI) is the only instrument currently in use that allows for severity classification: no insomnia (score 0–7), subclinical insomnia (score 8–14), or moderate to severe insomnia (score 15–28) (6).

The characterization of different phenotypes is important to establish clinically relevant subtypes of insomnia. It may help to reduce the heterogeneity of insomnia and facilitate cause identification and personalized treatments. Yet there are not many standardized instruments of insomnia diagnosis allowing for phenotyping. However, there has been evidence that insomniacs with a total sleep time of <6 h suffer a more severe insomnia than insomniacs with a total sleep time of 6 h or more (7). They display mental and psychological impairment compared to patients with average or longer than average sleep. However, mortality is increased for insomniacs with longer total sleep time (8). The sleep duration with the 6-h distinction also influences the therapy outcome, success of cognitive-behavioral therapy (CBT), and the relation to comorbid bipolar disorder (9, 10). Recently, a study investigated subtypes of insomnia according to psychological stress (11). Questioning 2,224 volunteers with an ISI score of at least 10 and a control group of 2,098 volunteers with an ISI score below 10, five insomnia subtypes were identified: highly distressed, moderately distressed but sensitive to positive reinforcement (accepting of positive emotions), moderately distressed insensitive to positive reinforcement, slightly distressed with a high reactivity to their environment and life circumstances, and slightly distressed with low reactivity. The results showed a high stability of the classification over the 5-year investigation. The psychological categorization is clinically relevant as there were clear differences identified between the subtypes regarding development, therapy success, presence of electroencephalogram (EEG) biomarker, and the risk for depression. This was a first approach to subtyping insomnia patients according to psychological health. The exact effect of psychological health, family history, comorbidity, personality, environment and sleep quality on insomnia is still unclear. Similar symptom clusters have been discussed for other disorders including depression (12).

Our study is part of the specific Research Topic introduced by Frontiers on the heterogeneity of insomnia and its comorbidity. We aim to encourage and further the discussion on insomnia heterogeneity and the need for possible phenotyping, we do not intend to provide a complete list of phenotypes or possible clusters. The study picked up the approach of subtyping insomnia by collecting a short questionnaire during anamnesis on possible related symptoms, onset and course of insomnia. We described phenotypical traits of insomniacs with a cohort of sleep disturbed patients from a specialized outpatient clinic for sleep disorders.

## Methods

### Participants and Recruitment

**Figure 1:**
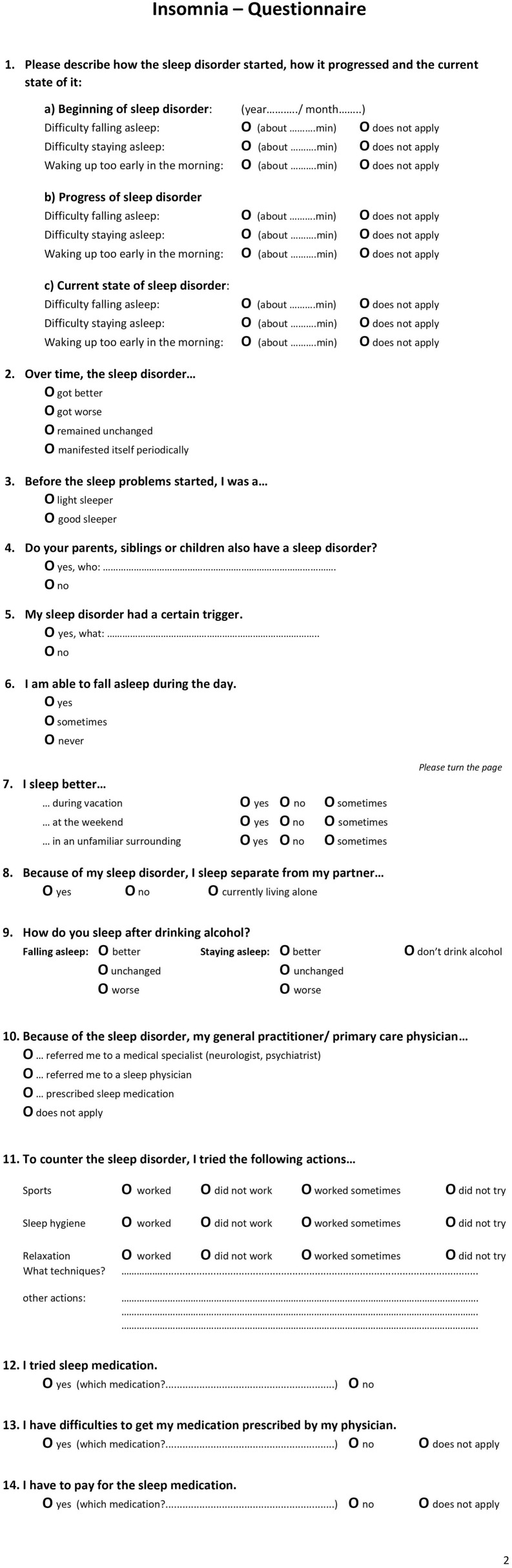
The English translation of the 14-item Insomnia Questionnaire with page 1 and page 2.

Since 2018, a specialized 14-item insomnia questionnaire has formed part of the clinical routine at the outpatient clinic of the Interdisciplinary Center of Sleep Medicine, Charité—Universitätsmedizin Berlin (Figure 1). The questionnaire is the result of literature research, clinical experience, and consensus of psychologists, neurologist, psychiatrists, and sleep physicians within the sleep center. Patients who visited the outpatient clinic between 01/2019 and 02/2020 and indicated self-reported symptoms presenting a suspicion of insomnia (e.g., difficulties initiating sleep, maintaining sleep, or early morning awakening) according to ICSD-3 criteria were recruited and completed the questionnaire. In total, 486 patients were examined by a physician specializing in sleep disorders and insomnia who confirmed an insomnia diagnosis. The questionnaire did not contain any identifying information. As the questionnaire is part of the clinical routine and the de-identified data has been analyzed retrospectively, ethical review and approval was not required in accordance with the local legislation and institutional requirements. As part of the clinical routine, patients signed informed consent forms allowing de-identified data of their patient file, including the insomnia questionnaire, to be used for research purposes.

### Questionnaire

The insomnia questionnaire consisted of 14 items (Figure 1 presents an English translation of the questionnaire). These included questions related to (1) type of insomnia (SOI—sleep onset insomnia, SMI—sleep maintenance insomnia, EMA—early awakening insomnia, multiple answers possible) at three points in time (start of disorder, progression, current state), (2) progression of insomnia, (3) sleep history of being a light or good sleeper, (4) relatives with sleep disorder, (5) triggers, (6) daytime sleep, (7) sleeping in different environments, (8) sleeping arrangement with partner, (9) alcohol as a sleep aid, (10) referral/ recommendation of general practitioner (multiple answer options), (11) alternative sleep treatments, and (12–14) sleep medication.

### Procedure

Procedure of the examination was standardized and performed by the same physician: On arrival, patients received several sleep questionnaires including the 14-item insomnia questionnaire. They were asked to complete these questionnaires before seeing the physician. During the following in-person consultation, the physician completed a full anamnesis (a patient-reported medical history) and confirmed a diagnosis of a primary insomnia according to ICSD-3 criteria. Next, the questions of the insomnia questionnaire were evaluated. Certain questions were clarified, and missing information added. For example, for question 3, light sleeper was defined. Light sleeper includes patients with a regular bedtime but whose sleep is sensitive to light, temperature, and noise. They need a specific degree of sleep comfort and sleep worse in an unfamiliar environment. These patients can nap during the day and sleep better during vacation and time off (e.g., weekends). They perceive their sleep as non-restorative. They also do not meet the diagnostic criteria of insomnia. The question refers to the time before the insomnia started, mostly referring to childhood / adolescence. For question 6, it was clarified that daytime napping included a daytime situation that explicitly allows for napping. For question 7, it was explained that “weekend” also included the days off work.

### Statistics

Sample size was calculated based on prevalence data and the estimated number of insomnia patients: ca. 30–50% of 328.2 million people (US population estimate 2019) result in about 98.5–164.1 million patients (13). With an accepted error rate of maximum 5% and a confidence interval of 95%, the sample size was set to at least 400 questionnaires in order to detect sufficiently powerful effects. Statistical analysis was performed using SPSS (IBM SPSS Statistics, Version 20). The patient cohort was described using a descriptive analysis with numbers and percentages (Table 1). In order to investigate possible insomnia subgroups based on phenotypes/characteristics, we compared items with dichotomous answers. Item 7 (sleeping in different environments), item 9 (alcohol as a sleep aid), and item 11 (alternative sleep treatments) each had several subcategories which were consolidated into one overall category. For the text answer of item 5 (trigger) we performed a qualitative data analysis by subjectively grouping the text data and visually presenting the categories. A t-test was used for group comparisons of continuous variables (e.g., age), the chi-square test for dichotomous variables. Significance level was set at 0.05.

**Table 1:** Sample description (*n* = 456 patients).

**Variable**	**Answers**	***n* (%)** ** (unless otherwise specified)**
Age (years)	M (SD) Mdn (IQR) range (min; max)	52.0 (15.9) 53.0 (39; 64) 68 (18; 86)
ISI	M (SD) Mdn (IQR) range (min; max)	18.4 (4.7) 19.0 (15.3; 21.0) 31 (1; 32)
Stop-Bang Questionnaire	M (SD) Mdn (IQR) range (min; max)	2.2 (1.4) 2.0 (1.0; 3.0) 7 (0; 7)
BDL-II	M (SD) Mdn (IQR) range (min; max)	13.4 (9.0) 12.0 (7.0; 18.0) 45 (0; 45)
RLS-DI	M (SD) Mdn (IQR) range (min; max)	2.1 (3.8) (0.0; 3.0) 36 (0; 36)
Duration of Insomnia (years)	M (SD) Mdn (IQR) range (min; max)	11.6 (10.9) 8.0 (3; 15) 82 (0; 82)
Gender	men women	199 (43.6%) 257 (56.4%)
Referral from general physician to *(multiple answers possible)*	specialist sleep physician sleep medication not applicable	118 (25.9%) 229 (50.2%) 159 (34.9%) 143 (31.3%)
Sleep medication	yes no no answer	316 (69.3%) 102 (22.4%) 38 (8.3%)
Sleeping separate from partner	yes no living alone no answer	106 (23.2%) 179 (39.3) 136 (29.8) 35 (7.7%)
Sleep history (before begin of the insomnia)	light sleeper good sleeper no answer	218 (47.8%) 196 (43.0%) 42 (9.2%)
Family with insomnia	yes no no answer	194 (42.5%) 222 (48.7%) 40 (8.8%)
Daytime sleep possible	yes sometimes no no answer	89 (19.5%) 202 (44.3%) 156 (34.2%) 9 (2.0%)
Trigger for insomnia	yes no no answer	194 (42.5%) 199 (43.6%) 63 (13.8%)
Different sleep environment helpful^*^	yes sometimes no no answer	120 (26.3%) 129 (28.3%) 170 (37.3%) 37 (8.1%)
Alternative sleep treatment helpful^§^	yes sometimes no not applicable	40 (8.8%) 190 (41.7%) 149 (32.7%) 77 (16.9%)
Alcohol as sleep aid helpful^$^	yes sometimes	71 (15.6%) 49 (10.7%)
	no no answer	170 (37.3%) 166 (36.4%)

*For “Age”, “ISI” (Insomnia Severity Index), “Stop-Bang Questionnaire”, “BDI-II” (Becks-Depression-Inventar Revision)”, RLS-DI” (Restless-Legs-Diagnose-Index), and “Duration of Insomnia”, mean (M), standard deviation (SD), median (Mdn), interquartile range (IQR), range with minimum (min) and maximum (max) are displayed. For all others, numbers (n) and percentages (%) are displayed*.

* *Different sleep environment included sleep on weekends, on vacation or different environment*.

§ *Alternative sleep treatment included sleep hygiene, sport, relaxation exercises*.

$ *Alcohol as sleep aid includes sleep onset and sleep maintenance*.

## Results

### Patient Description

Due to missing information that could also not be completed during the in-person consultation with the physician, 30 questionnaires were removed from analysis. The remaining 456 questionnaires were de-identified and analyzed. The patient cohort (Table 1) reported having sleep problems for an average of 11.6 ± 10.9 years (range: 0–82 years, where 0 means the symptoms just started within the past month). The cohort consisted of slightly more female insomniacs (56%) and had an average age of 52.0 ± 15.9 years (range: 18–86 years). More than half of the patients reported having a partner and not living alone (63%), and of those 37% slept in a separate room due to the sleep disorder. If the patient went to a general physician first, 50% were referred to a sleep specialist and 26% to another specialist (neurologist, psychiatrist etc.). In 35% of those cases, the general physician initiated a therapy with sleep medication. In general, 69% of the patients reported having used sleep medication, 23% indicated that they had not. Only 9% mentioned that it was difficult to get sleep medication. While 26% stated they had to pay for sleep medication, 37% said they did not. In Germany, sleep medication for primary insomnia covered by insurance only includes melatonin (only for patients over 55 years) and z-drugs (only for the acute therapy of 4 weeks).

### Sleep Characteristics

**Figure 2:**
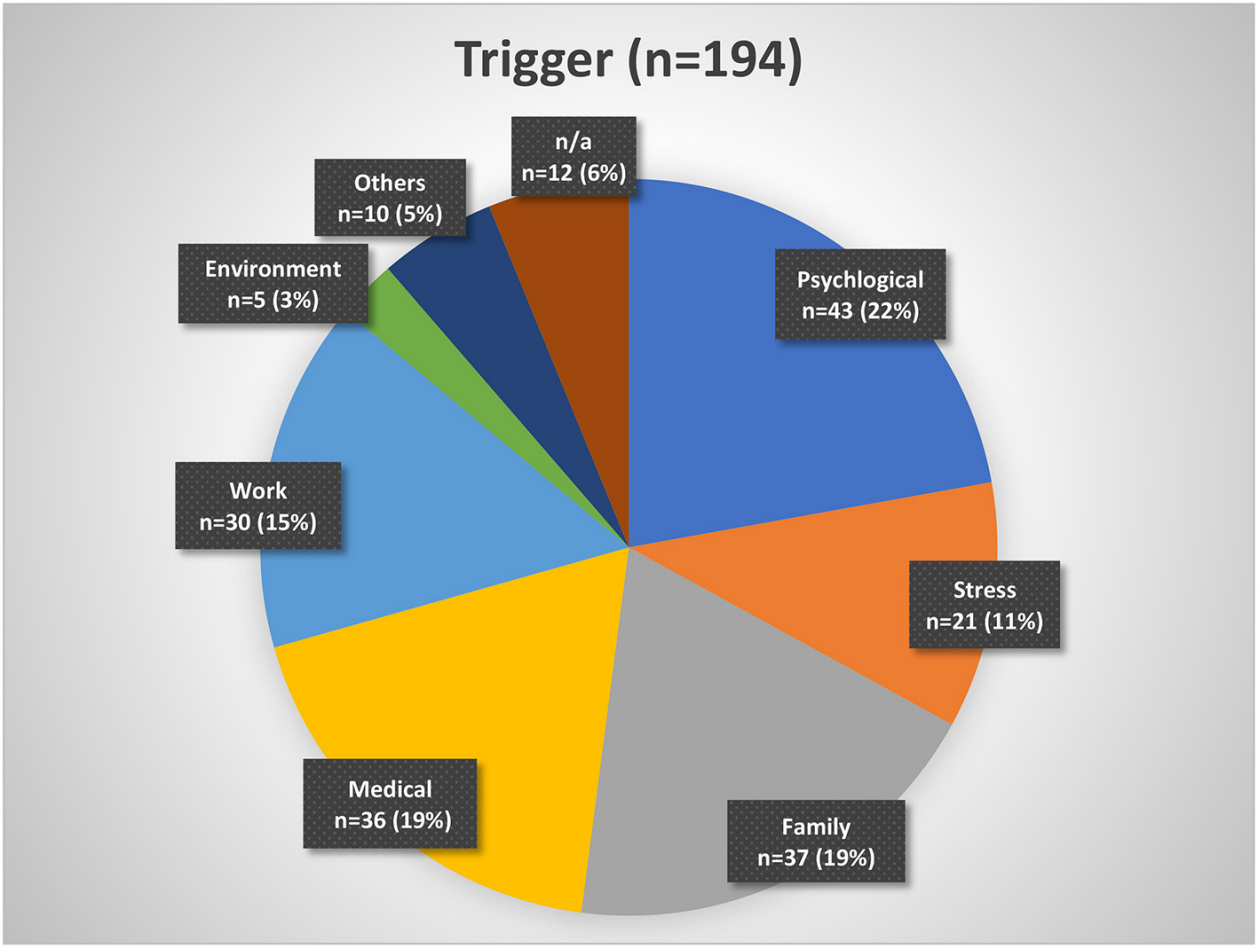
Insomnia triggers organized by categories. Psychological triggers include depression, fear, trauma, etc. Stress may be considered a subgroup of psychological triggers. Family triggers include birth, children, marriage, divorce, etc. Medical triggers include sickness, operations, etc. Work triggers include mobbing, loss of job, change of job, workload, etc. Environment triggers include noise, lighting, neighborhood, etc. Other triggers include smoking, attitude, etc. n/a, not available.

About 43% of the patients indicated that they had a history of being good sleepers before the insomnia onset, while 48% mentioned that they have always been light sleepers. Forty-three percent reported having a family member with sleep problems. Despite insomnia symptoms, 20% of patients indicated that they were able to fall asleep during the day and 44% sometimes. While 43% of patients reported a trigger for the sleep problems, 42% reported no trigger (Table 1). Figure 2 presents a categorization of the reported triggers. The most frequent triggers were of psychological nature (22%) including depression, anxiety, post-traumatic stress disorder, death of a family member, trauma, rape, psychotherapy etc. Stress was listed as a separate category but is to be considered as a subcategory of psychological triggers (additional 11%). Work related triggers including change or loss of job, freelance work, work problems, shift work, long work hours, workload, mobbing/ bulling etc. accounted for 15%.

The question about sleep in a different environment (item 7 of the questionnaire) included three subcategories: sleep during vacation, sleep at weekends, and sleep in unfamiliar surroundings. Sleep during vacation was perceived as better by 21% (*n* = 84), sometimes better by 30% (*n* = 121), and not at all better by 49% (*n* = 198). Sleep at the weekend was perceived as better by 18% (*n* = 70), sometimes better by 26% (*n* = 103), and not at all better by 56% (*n* = 224). Sleep in unfamiliar surroundings was perceived as better by 5% (*n* = 19), sometimes better by 17% (*n* = 68), and not at all better by 78% (*n* = 304). We consolidated the subcategories in one general environment variable. First, sleep in a different environment (in general) was considered better if a patient answered “yes (sleep better)” to at least one of the subgroups. The remaining patients were categorized into the sometimes group if they answered “sometimes” to at least one of the subcategories. Then, the remaining patients were categorized into the “no (do not sleep better)” or “no answer” category. In general, 26% indicated that they sleep better in different environments, 28% sometimes, and 37% not at all (Table 1).

The question for alternative non-medical treatments (item 11) also included three subcategories: sport, sleep hygiene, and relaxation techniques. Sport only helped in 7% (*n* = 26), helped sometimes in 32% (*n* = 130), and did not help in 46% (*n* = 185). Sleep hygiene helped in 5% (*n* = 18), helped sometimes in 29% (*n* = 103), and did not help in 43% (*n* = 154). Relaxation techniques helped in 5% (*n* = 19), helped sometimes in 32% (*n* = 117), and did not help in 38% (*n* = 142). We combined the subcategories into one overall variable of non-medical treatment in the same way as for item 7. In general, 9% of the patients indicated that an alternative treatment helps, 42% mentioned it helped sometimes, and 33% reported it did not help at all (Table 1).

Alcohol as a sleep aid (item 9) included two subcategories: alcohol as a sleep aid for sleep onset and alcohol as a sleep aid for sleep maintenance. While 40% (*n* = 112) indicated alcohol helps with SOI symptoms, it did not change sleep onset in 41% (*n* = 116) and symptoms got worse in 19% (*n* = 54). Alcohol helped with SMI symptoms in 11% (*n* = 31), did nothing in 46% (*n* = 123), and got worse in 43% (*n* = 116). We also consolidated this variable. Alcohol as a sleep aid in general helped, if a patient answered “sleep got better” to at least one of the two subcategories (without a “sleep got worse” for the other category). Alcohol worsened sleep if a patient answered at least once “got worse” (without a “got better” for the other category). We added the answer option “alcohol helps sometimes” for patients that answered “got better” to one of the categories and “got worse” to the other. The remaining patients were categorized as “no change” or “no answer.” In general, alcohol helped in 16%, helped sometimes in 11%, and did not help (or even got worse) in 37% (Table 1).

Table 2 presents a further description of insomnia subtypes based on these sleep characteristics. We dichotomized the answers into yes/no in order to create a more equal group distribution for comparison. Patients with a sleep history of being light sleepers even before insomnia onset, had significantly longer insomnia symptoms than patients with a sleep history of being good sleepers (*p* < 0.05). Patients with a family history of sleep problems were significantly more frequently female (*p* < 0.05), had suffered from insomnia symptoms significantly longer (*p* < 0.01), and presented significantly more EMA symptoms (*p* < 0.05) than patients without a family history of sleep problems. Patients who were able to sleep during the day were significantly more frequently male (*p* = 0.001) and displayed fewer SOI (*p* < 0.001) and fewer EMA symptoms (*p* < 0.01) than patients who could not sleep during the day. Patients with no trigger displayed a tendency to having a longer insomnia duration than patients with a trigger (*p* = 0.05). Patients who were able to sleep better in different environments were significantly younger (*p* < 0.001) and showed a tendency to shorter insomnia duration (*p* = 0.05) than patients who did not sleep better in another environment. Patients for whom alcohol helped as a sleep aid were significantly younger (*p* < 0.001) and presented significantly more SOI symptoms (*p* < 0.001).

**Table 2:** Description of possible insomnia phenotype subgroups based on sleep characteristics.

**Sleep Characteristics**	**Age** ** (years)**	**Men**	**Duration of** ** Insomnia** ** (years)**	**Patients** ** with SOI** ** symptoms**	**Patients** ** with SMI** ** symptoms**	**Patients** ** with EMA** ** symptoms**
	**M±SD**	***n* (%)**	**M±SD**	***n* (%)**	***n* (%)**	***n* (%)**
**Sleep history**						
Light sleeper (*n =* 218)	51.7 ± 15.8	89 (40.8%)	11.8 ± 10.9	131 (60.1%)	152 (69.7%)	113 (51.8%)
Good sleeper (*n =* 196)	52.3 ± 15.9	92 (46.9%)	9.5 ± 7.7	99 (50.5%)	133 (67.9%)	84 (42.9%)
*p*	0.682	0.211	**0.022**	0.065	0.646	0.093
**Family with sleep problems/insomnia**						
Yes (*n =* 194)	49.8 ± 15.4	73 (37.6%)	13.4 ± 12.9	111 (57.2%)	135 (69.6%)	99 (51.0%)
No (*n =* 222)	52.7 ± 16.3	105 (47.3%)	10.0 ± 8.8	119 (53.6%)	143 (64.4%)	93 (41.9%)
*p*	0.061	**0.047**	**0.005**	0.670	0.097	**0.028**
**Daytime sleep possible**						
Yes (*n =* 291)	52.3 ± 16.5	143 (49.1%)	11.8 ± 11.7	140 (48.1%)	194 (66.7%)	119 (40.9%)
No (*n =* 156)	51.3 ± 15.0	52 (33.3%)	11.4 ± 9.6	107 (68.6%)	107 (68.6%)	88 (56.4%)
*p*	0.567	**0.001**	0.777	**0.000**	0.176	**0.004**
**Trigger**						
Yes (*n =* 194)	52.3 ± 15.3	75 (38.7%)	10.3 ± 10.0	110 (56.7%)	125 (64.4%)	88 (45.5%)
No (*n =* 199)	51.4 ± 16.9	93 (46.7%)	12.7 ± 11.8	105 (52.8%)	141 (70.9%)	96 (48.2%)
*p*	0.576	0.106	0.054	0.217	0.466	0.349
Different environment helpful^*^						
Yes (*n =* 249)	47.9 ± 15.1	112 (45.0%)	11.0 ± 9.4	143 (57.4%)	166 (66.7%)	129 (51.8%)
No (*n =* 170)	57.0 ± 14.9	77 (45.3%)	13.6 ± 13.4	90 (52.9%)	116 (68.2%)	64 (37.6%)
*p*	**0.000**	0.949	0.053	0.689	0.427	0.115
**Alternative treatment helpful^§^**						
Yes (*n =* 230)	49.6 ± 15.2	101 (43.9%)	11.8 ± 11.0	134 (58.3%)	162 (70.4%)	119 (51.7%)
No (*n =* 149)	52.5 ± 16.6	56 (37.6%)	12.7 ± 11.9	86 (57.7%)	98 (65.8%)	65 (43.6%)
*p*	0.083	0.222	0.509	0.520	0.717	0.604
**Alcohol as sleep aid helpful^$^**						
Yes (*n =* 120)	46.7 ± 15.7	59 (49.2%)	12.2 ± 10.4	84 (70.0%)	80 (66.7%)	63 (52.5%)
No (*n =* 170)	53.1 ± 14.3	74 (43.5%)	11.4 ± 10.2	73 (42.9%)	113 (66.5%)	77 (45.3%)
*p*	**0.000**	0.343	0.540	**0.000**	0.269	0.887

* *Different sleep environment included sleep on weekends, on vacation or different environment*.

§ *Alternative sleep treatment included sleep hygiene, sport, relaxation exercises*.

$ *Alcohol as sleep aid includes sleep onset and sleep maintenance*.

*SOI, Sleep onset insomnia; SMI, sleep maintenance insomnia; EMA, early morning awakenings insomnia. Symptoms are not exclusive; they can occur either as single symptom or in occurrence with other symptoms. M, mean; SD, standard deviation; n, number. The % refers to the corresponding answer category as a base, listed in the left row. For group comparisons, p was calculated with a t-Test for the continuous variable age and with chi-square tests for the dichotomous variables. Significance level was set at 0.05. Significant differences were highlighted*.

### Insomnia Symptom Subtypes and Progression

At time of visit, 54% of patients presented SOI symptoms, 66% SMI symptoms, and 45% EMA symptoms (Table 3). In 57% of the patients, there was a combination of those symptoms. Patients with SOI symptoms reported on average that they needed 85.6 ± 55.0 min to fall asleep. Patients with SMI symptoms reported waking up for about 79.0 ± 58.2 min after sleep onset. And patients with EMA symptoms reported that they woke up on average 79.0 ± 56.5 min too early in the morning. Patients with EMA symptoms (not exclusively, combination of symptoms possible) had the shortest history of sleep problems (10.2 ± 9.1 years, range: 0–44 years) compared to patients with SOI symptoms (12.0 ± 9.8 years, range: 0–82 years) and patients with SMI symptoms (11.5 ± 10.6 years, range: 0–82 years). Differences were not significant.

**Table 3:** Patient description by insomnia subgroups based on symptoms over time.

	**Begin of** ** Insomnia**	**Current** ** Status**	***P***
**SOI symptoms:** ***n*** **(%, of all patients)**	260 (57.0%)	248 (54.4%)	0.634
Age in years: M±SD	50.7 ± 16.6	50.5 ± 16.3	0.883
Men: *n* (%, of SOI)	104 (40.0%)	99 (39.9%)	0.985
Single symptom: *n* (%, of SOI)	68 (26.2%)	47 (19.0%)	0.053
Symptom Combination: *n* (%, of SOI)	192 (73.8%)	201 (81.0%)	0.053
**SMI symptoms: n (%, of all patients)**	302 (66.2%)	303 (66.4%)	0.258
Age in years: M±SD	53.1 ± 15.6	52.3 ± 15.7	0.520
Men: *n* (%, of SMI)	131 (43.4%)	134 (44.2%)	0.834
Single symptom: *n* (%, of SMI)	65 (21.5%)	51 (16.8%)	0.149
Symptom Combination: *n* (%, of SMI)	237 (78.5%)	252 (83.2%)	0.149
**EMA symptoms:** ***n*** **(%, of all patients)**	184 (40.4%)	207 (45.4%)	**0.016**
Age in years: M±SD	50.9 ± 15.6	49.7 ± 15.4	0.452
Men: *n* (%, of ESA)	75 (40.8%)	87 (42%)	0.799
Single symptom: *n* (%, of ESA)	8 (4.3%)	11 (5.3%)	0.657
Symptom Combination: *n* (%, of ESA)	176 (95.7%)	196 (94.7%)	0.657

*SOI, Sleep onset insomnia; SMI, sleep maintenance insomnia; EMA, early morning awakenings insomnia. Patients were divided into subgroups of insomnia symptoms. Symptoms are not exclusive; they can occur either as single symptom or in occurrence with other symptoms. Symptoms were assessed for two times: At begin of insomnia and current state. For comparisons over time, p was calculated with chi-square tests. Significance level was set at 0.05. Significant differences were highlighted*.

**Figure 3:**
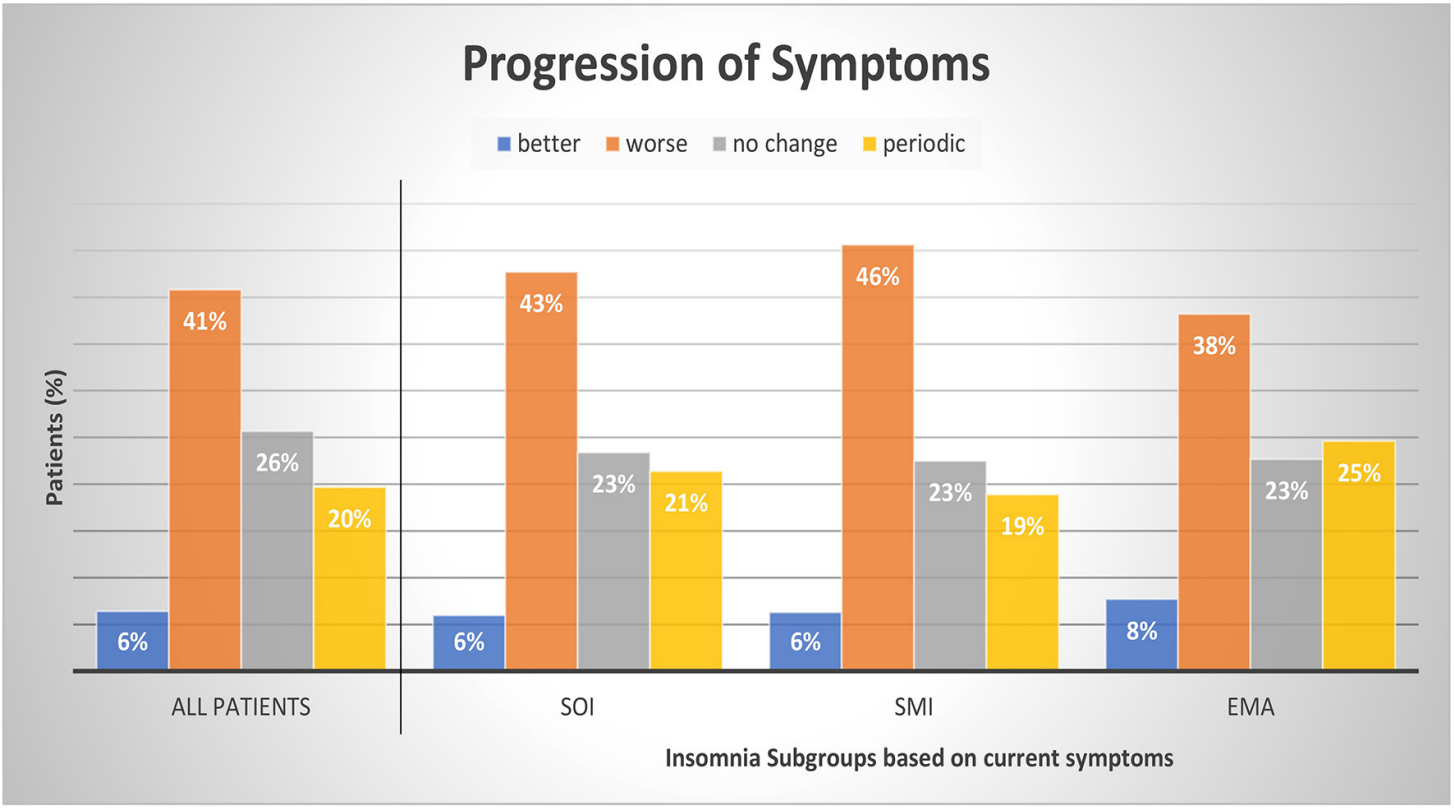
Progression of symptoms by insomnia subgroups. Patients were divided into subgroups of current insomnia symptom. Symptoms are not exclusive, they can occur either as single symptom or in occurrence with other symptoms. SOI, Sleep onset insomnia; SMI, sleep maintenance insomnia; EMA, early morning awakenings insomnia. A patient with a periodic pattern of insomnia experiences weeks or months long periods with insomnia symptoms alternating with symptom free periods. For comparisons between symptom groups, *p* was calculated with chi-square tests. Results were not significant at a 0.05 level. The sum of the subcategories does not add up to 100% as we refrained from displaying the category “missing data and multiple answers” (7% All patients, 7% SOI, 6% SMI, and 7% EMA).

Table 3 presents the possible change of sleep symptoms over time by type of sleep symptoms. There was no significant change in SOI or SMI symptoms. Only EMA symptoms significantly increased over time (*p* = 0.016). Figure 3 presents the progression in severity of the sleep disorder. Fewer than 10% reported an improvement of symptoms, while in 41% the sleep disorder got worse. In 20% the symptoms showed a periodic pattern. The progression was independent of current symptoms.

## Discussion

A distinct cohort of insomnia patients that reported to a special outpatient clinic for sleep disorders revealed that about 40–50% of the patients mentioned a trigger for the sleep problems, were not good sleepers to begin with (light sleepers), had a family history of sleep problems, and had a progressive course of insomnia. Over one third were not able to fall asleep during the day. Insomnia with SMI symptoms was most frequent, as well as a psychological trigger. Over time, EMA symptoms increased. Alternative non-medical treatments were only lastingly effective in fewer than 10%. Over two thirds of the patients (69%) had tried sleep medication. One of the unique traits of our cohort is the duration of the sleep problem before the visit to a specialist (over 11 years). For most, the sleep specialist/clinic is not the first point of contact. Thus, our patient cohort is not comparable to one from a general physician or population-based cohort.

Our results emphasize the insomnia heterogeneity and the need for phenotyping. Following, we will first discuss the characteristics assessed with our questionnaire starting with some new aspects that are currently not commonly asked (history of being a light sleeper, daytime sleep, effects of alternative treatments, alcohol, temporal stability/change of insomnia symptoms). Then, we will review the current literature for further possible phenotypes. Table 4 presents an overview.

**Table 4:** Overview of discussed phenotypes.

**Phenotypes (in our cohort)**	**In our cohort**	Comments/Literature^*^
Sleep history (light sleeper)	48%	**Relatively new aspect** (14)
Daytime sleep (not possible)	34%	**Relatively new aspect** (15)
Alternative treatment (positive effect)	51%	**Relatively new aspect** (9)
Alcohol (positive effect)	26% (40% with sleep onset, 11% with sleep maintenance)	**Relatively new aspect** (16)
Insomnia onset (SMI symptoms)	66%	**Relatively new aspect** (16)
Symptom stability (EMA symptoms increase)	From 40 to 45%	**Relatively new aspect** (17)
Family history with insomnia (yes)	43%	(18–21)
Trigger (yes)	43%	(22–24)
Progression of insomnia (negative)	41%	(25–29)
Sleeping in different environments (better)	54%	(30, 31)
**Other Phenotypes (not recorded in our cohort)**		**Comments/Literature**
Life history (including trauma and life events) as trigger, Comorbidities (other sleep disorders, depression, anxiety, etc.), Chronotype, Mood, Quality of life, Personality, Sensitivity, Dysfunctional beliefs, Emotion regulation, and more		(32) (mentioned 17 characteristics to be asked during anamnesis, a complete list is mentioned in the discussion)
Non-sleep phenotypes (e.g., life history, mood, personality, etc.)		(11)
Biomarkers of EEG, pulse rate, heart rate variability, etc.		(33, 34)
Age at onset, Time to see a specialist, Frequent nocturnal awakenings, Type of insomnia onset (suddenly vs. slowly)		(35, 36) (Sleep Condition Indicator)

*SOI, Sleep onset insomnia; SMI, sleep maintenance insomnia; EEG, electroencephalogram*.

* *The literature mentioned is not the result of a systematic review analysis. However, phenotypes with only one literature mentioned indicate that there are only few literature to be found. n/a, not applicable as not recorded in our cohort*.

### Phenotypes—Based on Our Cohort

#### Sleep History

Almost half of our cohort (48%) presented a bad sleep history, indicative of an idiopathic insomnia.

There are no clear biomarkers or diagnostic criteria to distinguish between psychophysiological and idiopathic (chronic) insomnia (14). In order to identify idiopathic insomnia, we ask the patient for their sleep history, specifically before insomnia onset. Did the patient always experience poor (light) sleep, or were they a fairly good sleeper? We assume that light sleep is the pre-stage of insomnia, but not every light sleeper needs to develop insomnia, indicating that these variables are not predictors for differentiating between psychophysiological and idiopathic insomnia. Whether this distinction of good and bad sleep before developing insomnia influences therapy will need to be further investigated. Also, the term “light (bad)” sleep needs to be clearly defined and standardized.

#### Daytime Sleep

Using our questionnaire, we found in our cohort that 34% of patients reported not being able to take a nap during the daytime despite being tired and despite having the explicit opportunity of taking a nap. Those patients were predominantly women with more SOI and more EMA symptoms compared to patients who were able to fall asleep during the day. They did not differ regarding the duration of their insomnia symptoms.

Currently, it is not common during insomnia diagnosis to ask whether a patient is able to fall asleep during the day or to conduct a Multiple Sleep Latency Test (MSLT) for objective assessment. Our own experience with insomnia patients, however, showed how important this question is. We experienced that patients who sleep poorly at night and are tired during the day, but cannot sleep in the day either, usually have a higher degree of insomnia. They tend to suffer for more nights a week and are more resistant to therapy. In contrast, the possibility of falling asleep during the day, in front of the television, in the car, on public transport, in a meeting, or in other quiet surroundings, seems to be a sign of a lower degree of insomnia.

The ability to nap during the day has also been a criterion for other indications in the literature. The Hyperarousal Scale by Regestein et al. (37) provides indirectly a reference to the degree of alertness during the day and thus to the inability to fall asleep. Khassawneh et al. (38) used the scale together with the patient's subjective statement that they cannot nap during the day and found that patients with hyperarousal and short sleep duration have more cognitive deficits in memory tests. Li et al. (39) used the MSLT with a threshold value of 14 min to define hyperarousal. Drake et al. (40) also used the MSLT and investigated sleep disturbances due to commonly experienced stressful situations to identify factors representing the construct of “stress-related” vulnerability to sleep disturbance. Subjects with a high Ford Insomnia Response to Stress Test (FIRST) score had poorer sleep quality at night and higher latencies of sleep in the MSLT. Roehrs et al. (15) performed the MSLT in 95 patients with primary insomnia (32–64 years) and in 55 healthy sleepers and found a higher sleep latency in insomniacs (13.2 ± 4.65 min vs. 11.0 ± 4.93 min). However, the difference is small and the variability among insomniacs is high (between 2 and 20 min). The MSLT is still a questionable method for diagnosing insomnia, but it may be a possible tool for subtyping insomnia with regard to the ability to fall asleep during daytime. Espie et al. (41) examined daytime symptoms of 11,129 participants with (*n* = 5,083) and without insomnia, coming from different backgrounds. Of the analyzed items (energy, concentration, relationships, ability to stay awake, mood, and ability to get through work), the items “energy” and “mood” turned out to be the two most important parameters for insomniacs, but not the item “ability to stay awake.” The importance of the criterion daytime sleepiness and/or ability to stay awake seems therefore recognized, but not yet uniformly defined and requires further research.

#### Alternative Treatment (Behavioral Therapy)

In our cohort, about 83% of the patients have tried at least one of these alternative non-medical behavioral treatments: sport, sleep hygiene, and/or relaxation techniques. In one third of the patients (33%) these techniques did not help. There were no significant age, gender, or symptom differences between patients with effective alternative treatments and patients where it was not effective. However, we did not investigate the severity of insomnia and it may be possible that patients where the alternative treatments did not show a positive effect may be patients with more severe insomnia.

Therapy recommendations for insomnia include a multi-modal behavioral therapy including psychological elements (e.g., CBT) as the first therapeutic step which many patients do complete, most commonly even before they arrange a visit to a specialist (42). This is also what we found in our cohort. Most of our patients have tried to educate themselves on their sleep problems, have tried to improve their sleep hygiene, have tried alternative non-medical treatments (e.g., sport, relaxation, etc.), and already went to either a natural health practitioner, homeopath, psychologist or psychotherapist. Currently, CBT is not yet good enough established in Germany as a definite treatment for insomnia. Studies have shown that CBT had less of an effect on insomniacs with short sleep duration (9). We assume that this also applies to patients with a more severe insomnia. However, severity has yet to been clearly defined. Patients will most likely show a similar reaction to phytopharmacology or alternative “smart” therapy (e.g., acoustic or electrical stimulation). A future quality check and standardization of CBT methods may be helpful in order to use the success of alternative treatment/behavioral therapy as a phenotypical criterion. We hypothesize that successful CBT is mainly linked to mild insomnia. For moderate to severe insomnia, CBT should be a necessary concomitant therapy.

#### Alcohol

In our cohort, only about 26% mentioned that alcohol helps with sleep problems in general. Patients for whom alcohol helped were significantly younger and presented more SOI symptoms. A more detailed analysis showed that alcohol helped especially with sleep onset (40%), less with sleep maintenance (only 11%). In 43% of our patients, alcohol even worsened sleep maintenance, which other studies confirmed (16). However, in almost half of our patients, alcohol showed no change.

Alcohol is a widely used sleep aid. Asking for the soporific effect of alcohol should become standard during insomnia anamnesis, as well as asking for the soporific effect of drugs (CBD, cannabis, etc.) which have become more and more a topic of sleep research (43). It is surprising that in our cohort many patients reported a lack of positive effect of alcohol as a sleep aid. It may be that the alcohol amount consumed was not high enough, as we did not ask for specifics.

#### Symptoms at Time of Insomnia Onset

In our cohort, 57% had SOI symptoms when the insomnia started (in 74% as a combination with other symptoms), 66% had SMI symptoms at the beginning (in 79% as a combination of symptoms), and 40% started with EMA symptoms (in 96% with other symptoms). The majority had a combination of several symptoms. Hence, in most cases of insomnia the sleep disorder started with SMI symptoms (either as single symptom or in combination). We found that patients with single SOI or single EMA were significantly younger than patients with a SOI combination (single: age 47 ± 17 years, combination: age 52 ± 16 years; *p* < 0.01) or EMA combination (single: age 39 ± 13 years, combination: age 51 ± 15 years; *p* < 0.01), respectively.

Bjorøy et al. (16) also investigated subtypes of insomnia in an extensive web-based survey with 64,503 patients who had displayed insomnia for >6 months. Here, 60% of the younger insomniacs (on average 37 years) showed SOI symptoms, either as a combination with SMI and/or EMA symptoms or as a single symptom. Confirming our own results, Bjorøy et al. (44) also found that SOI as a single symptom was more frequent in younger insomniacs, a SOI symptom combination more frequent in older insomniacs. They revealed further predictors for a symptom combination including female gender, evening chronotype, less education, and being single. While we do not assess aspects such as chronotype, they are important. Literature has shown that there is a higher insomnia prevalence in general in people with an evening chronotype. Insomniacs with a symptom combination also showed a higher comorbidity with depression, anxiety, and a higher use of alcohol and sleeping pills (16).

#### Symptom Stability Over Time

Not just the severity, but also the symptoms can change over time. In our cohort, prevalence of SOI and SMI symptoms did not change; EMA symptoms, however, significantly increased from 40 to 45% from first noticing those symptoms to the present (visit to a sleep specialist). Patients with SOI symptoms showed a tendency of an increase of SOI in symptom combination instead of as a single symptom (from 74 to 81%).

An early study of Hohagen et al. (17) also investigated the progression of insomnia symptoms and possible temporal stability of different patterns in 328 patients (18–65 years). In only 4 months, they discovered a >50% change in SOI, SMI, and EMA symptoms. Only in rare cases did a specific and single symptom insomnia (either SOI, SMI, or EMA) change from one to another single symptom. However, in many single symptom insomnia cases another symptom occurred over time while the first symptom stayed dominant. This tendency was also seen in our cohort regarding the SOI symptoms.

#### Family History

Almost half of our patient cohort (43%) reported a family history of disturbed sleep/insomnia. These patients were foremost female and presented more EMA symptoms than patients without a family history present.

A specific gene for insomnia is not known but a genetic predisposition cannot be completely ruled out (18, 19). A twin study of children revealed a moderate inheritability of insomnia, and another study reported 35% inheritability (20, 21).

#### Trigger

In our cohort, almost every second patient (43%) reported a trigger. Patients with or without a trigger in our cohort did not differ regarding age, gender, and insomnia symptoms. However, those patients with no triggers showed a tendency to longer insomnia duration then the ones with a trigger. Here, it may be possible that the start of the trigger (whether sudden or slowly, unconsciously developing) may have an impact on the perception of insomnia as a chronic condition. Within our cohort, most frequently named were psychological triggers (e.g., depression, anxiety, trauma, burnout), family triggers (e.g., birth, divorce, custody battles), and medical/biological triggers including surgery and other illnesses. Work triggers (e.g., mobbing/ bulling, job loss) and stress as a separate psychological trigger came next.

Triggers are part of Spielman's theoretical model (1987) of factors causing chronic insomnia. The 3Ps consist of predisposing factors, precipitating factors which trigger acute insomnia, and perpetuating factors (22, 23). Triggers would belong to the precipitating factors and may lead to a chronic insomnia. For a working patient, work related stress and job strain may play a bigger role as a trigger and moderator of the insomnia than for those patients that are not working (24). However, whether the existence of a trigger influences the progression or therapy of insomnia still needs to be further investigated.

#### Progression of Insomnia

Our patients reported most frequently a negative progression of insomnia (41%); in 26% there were no changes, and only in 7% was there an improvement. On average, the patients suffered from insomnia symptoms for about 11.6 years (range 0–82 years) before seeing a sleep specialist. Patients with predominantly EMA symptoms showed the shortest sleep problem history with 10.2 years (range 0–44 years) compared to patients with SOI or SMI symptoms. About 20% of our patients reported a periodic pattern of symptom severity.

The periodic pattern may be indicative of a non-24 h disorder (25). A patient with a periodic pattern of insomnia experiences weeks or months long periods with insomnia symptoms alternating with symptom free periods. Green et al. (26) also investigated the progression of insomnia for over 20 years in 5-year intervals. Patterns included: healthy pattern, episodic pattern, chronic pattern, and a pattern with the development of symptoms in the follow-up period. Chronic insomnia was linked to older women and the working class. It showed that social factors do affect the progression of a sleep disorder, a fact also indicated by Patel et al. (27) and Arber et al. (28). There is another distinction of insomnia subtypes by progression introduced by Wu et al. (29): persistent insomnia, remission, or relapse.

#### Sleep in Different Environments

Over half of our patients (54%) reported sleeping better in a different environment, including weekends/days with time off from work (51%), vacation (44%), and unfamiliar surroundings in general (22%). The category “unfamiliar surroundings” received the lowest number. Patients may have included job related hotel stays and therefore increased stress level, which may account for the lower number. Patients stating they slept better in a different environment were predominantly younger members of our cohort.

If patients reported sleeping better at weekends or on vacation, this may be an indication that the sleep disorder was caused by work stress or daily routine. In the literature, this is called behavioral induced insufficient sleep (30, 31). As only few insomniacs are able to quit their job or family, this category may represent a specific insomnia phenotype. For those, specific interventions are possible including the end of shift work, change to home office work, change from full-time to part-time work, etc.

### Further Discussion of Phenotypes

Studies suggest that insomnia is a heterogenic disorder and the identification of different phenotypes or comorbidities is important for personalized treatments (45). In our study, we presented some new aspects on what insomniacs should be asked during anamnesis and what should be considered during phenotyping. Benjamin et al. (32) already proposed the following characteristics: (1) life history including demographics, mental and physical health, trauma and life events. This study showed that more women than men and more older people than younger people suffer from insomnia and life events are usually triggers. Such triggers are mostly to be found at home, in health or at work/school, as could also be confirmed with our patients. But who reacts to such a negative trigger with insomnia and why, when, at what age, is not yet known and may possibly have a genetic reason. Further characteristics included (2) subjective sleep quality, (3) fatigue, sleepiness, hyperarousal in the daytime, (4) other sleep disorders, (5) lifetime sleep history, (6) chronotype, (7) depression, anxiety, mood, (8) quality of life, (9) personality, (10) worry, rumination, self-consciousness, sensitivity, (11) dysfunctional beliefs, (12) self-conscious emotion regulation and coping, (13) nocturnal mentation, (14) wake resting state mentation, (15) lifestyle including physical activity and food intake, (16) body temperature, and (17) hedonic evaluation. Other possible non-sleep phenotypes included: MRI, cognition, mood, traits, history of life events, family history, PSG, sleep microstructure, genetics. Blanken et al. (11) distinguished insomnia subtypes according to the so-called non-sleep categories of life history, mood perception, and personality. Miller et al. (33) presented an insomnia cluster analysis based on neurocognitive performance, sleep-onset measures of qualitative EEG, and heart rate variability (HRV). They identified two main clusters, depending on duration of sleep (<6 h vs. >6 h). The HRV changes during falling asleep may also play a role, as may the spectral power of the sleep EEG, and parameters from the sleep hypnogram such as sleep onset latency and wake after sleep onset. In one of our own studies, we were able to demonstrate that the increased nocturnal pulse rate and vascular stiffness in insomniacs with low sleep efficiency (<80%) represented an early sign of elevated cardiovascular risk, and thus presented a useful tool for phenotyping insomnia (34). In the future, other objective characteristics may include biomarkers or radiological features (46, 47).

Further characteristics that may play a role but have not yet been mentioned or investigated are the age of the patient during insomnia onset, frequent nocturnal awakenings, the time it takes to see a specialist, and the kind of insomnia onset, slowly progressing or suddenly unexpected. There is no defined age at which the likelihood of insomnia increases, but we know that menopause is a major trigger for women. Grandner et al. (35) were able to show that getting older alone is not a predictor of insomnia, it rather includes multifactorial events. The question of how long it takes to see a specialist is also part of the Sleep Condition Indicator (SCI) by Espie et al. (36). They asked whether the insomnia had lasted longer than a year, 1–2, 3–6, or 7–12 months. We can easily agree with such a classification in terms of content. Many patients who wake up frequently at night consider this an insomnia with SMI symptoms. Frequent nocturnal awakenings, but with the ability to fall asleep again straight away, are according to the definition not considered a SMI insomnia. We did not address this in the present study, which presents a limitation. While it is mentioned in the DSM-5 as an independent sign of insomnia, patients affected by frequent nocturnal but subjectively normal sleep lengths and still restful sleep do not (yet) have insomnia. Whether it is an independent phenotype or a preliminary stage of a SMI insomnia should be further examined and defined. It also needs to be clarified whether devices for sleep registration help us with phenotyping. Polysomnography is certainly a very strong phenotypic feature when sleep time is very short, wake times after sleep onset is high and deep and/or dream sleep and sleep efficiency are not optimal. However, the current status is such that it is not suitable for diagnosis (48). In the near future, technical advances will help to provide objective, long-term sleep data, which are important for diagnosis, subtyping, and therapy for different types of insomnia.

Currently, questionnaires have been used to assess insomnia. The most known questionnaires include the ISI and the Pittsburgh Sleep Quality Index (PSQI). These are valid instruments (6, 49). However, there are a number of other questionnaires used for insomnia such as the Amsterdam Resting-State Questionnaire (ARSQ), Dysfunctional Beliefs and Attitudes About Sleep Scale (DBAS), Sleep-Related Behaviors Questionnaire (SRBQ), Sleep Functional Impact Scale (SFIS), Leeds Sleep Evaluation Questionnaire (LSEQ), Glasgow Sleep Effort Scale (GSES) (50–55). In 2014, Espie et al. (36) introduced the SCI which presented a good instrument for identifying the presence of insomnia and also allowed for time differentiation. Also, the short version with only 2 questions seems valid, where questions are asked about the number of nights in the past month with poor sleep and about the trouble in general caused by sleep (56). Kalmbach et al. (57) presented a differentiation between good and bad sleepers based on the Presleep Arousal Scale—Cognitive (PSAS-C) and—Somatic (PSAS-S). People with a high PSAS-C have higher sleep latency and wake times after sleep onset, as well as higher MSLT latency and lower sleep efficiency and total sleep time. The PSAS-C in particular seems to be a good measure of the hyperarousal state. Research and official expert recommendations will show which questionnaires should be favored in clinical practice.

### Limitations

Our study intended to encourage and further the discussion on insomnia heterogeneity and the need for possible phenotyping. While we introduced some new aspects of phenotyping, we neither provided a complete list of possible phenotypes nor defined specific clusters. Limitations of our study include the fact that further important aspects (e.g., comorbidity, employment, having children, chronotype, employment etc.) may need consideration. Also, some aspects of the questionnaire will need a more precise definition (e.g., light sleeper, daytime napping, weekend/vacation, alternative treatment, alcohol use), patients were not differentiated regarding sleep duration (<6 h vs. >6 h), and the progression of insomnia was observed retrospectively and not investigated prospectively. While our study was performed with patients of a sleep center, there is also need for phenotyping and thorough assessment of those phenotype characteristics in patients of a primary care setting.

## Conclusion

As part of a specific Research Topic introduced by Frontiers on the heterogeneity of insomnia, our study provides further ideas on the already existing approaches to phenotyping insomnia patients. The aim of our study was not to examine all conceivable phenotypic features of insomnia, but to help document specific characteristics with simple questions about the onset and course of insomnia during anamnesis. While the clinical relevance of some of those possible phenotypes is not yet clear (e.g., sleep history, trigger, daytime sleep, sleep in a different environment, alternative treatment, insomnia progression/symptom stability etc.), they should play a role in future research and medical care of insomnia patients. We would like to give an impulse for further research in this area, in order to better differentiate insomnia, thus leading to more effective individualized therapy.

## Data Availability Statement

The raw data supporting the conclusions of this article will be made available by the authors, without undue reservation.

## Ethics Statement

Ethical review and approval was not required for the study on human participants in accordance with the local legislation and institutional requirements. The patients/participants provided their written informed consent to participate in this study.

## Author Contributions

IF, TP, and VK had the role of supervision and conceptualized the study. IF was responsible for data collection. NL performed data analysis. All authors were involved in visualization and writing including data interpretation, result discussion, and drafting and reviewing the manuscript.

## Conflict of Interest

The authors declare that the research was conducted in the absence of any commercial or financial relationships that could be construed as a potential conflict of interest.

## References

[1] Ohayon MM. Epidemiology of insomnia: what we know and what we still need to learn. Sleep Med Rev. (2002) 6:97–111. 10.1053/smrv.2002.0186 12531146

[2] Morin CM LeBlanc M Daley M Gregoire JP Merette C. Epidemiology of insomnia: prevalence, self-help treatments, consultations, and determinants of help-seeking behaviors. Sleep Med. (2006) 7:123–30. 10.1016/j.sleep.2005.08.008 16459140

[3] Krystal AD Prather AA Ashbrook LH. The assessment and management of insomnia: an update. World Psychiatry. (2019) 18:337–52. 10.1002/wps.20674 31496087 PMC6732697

[4] American Academy of Sleep Medicine. International Classification of Sleep Disorders. 3rd ed. Darien, IL: American Academy of Sleep Medicine (2014).

[5] American Psychiatric Association. Diagnostic and Statistical Manual of Mental Disorders – Section II: Diagnostic Criteria and Codes: Sleep-Wake Disorders. 5th ed. Arlingten, VA: American Psychiatric Association (2013).

[6] Bastien CH Vallières A Morin CM. Validation of the nsomnia Severity Index as an outcome measure for insomnia research. Sleep Med. (2001) 2:297–307. 10.1016/S1389-9457(00)00065-4 11438246

[7] Vgontzas AN Fernandez-Mendoza J Liao D Bixler EO. Insomnia with objective short sleep duration: the most biologically severe phenotype of the disorder. Sleep Med Rev. (2013) 17:241–54. 10.1016/j.smrv.2012.09.005 23419741 PMC3672328

[8] Wallace ML Lee S Hall MH Stone KL Langsetmo L Redline S. Heightened sleep propensity: a novel and high-risk sleep health phenotype in older adults. Sleep Health. (2019) 5:630–8. 10.1016/j.sleh.2019.08.001 31678177 PMC6993140

[9] Bathgate CJ Edinger JD Krystal AD. Insomnia patients with objective short sleep duration have a blunted response to cognitive behavioral therapy for insomnia. Sleep. (2017) 40: zsw012. 10.1093/sleepj/zsx050.334 28364452 PMC6084751

[10] Lewis KJS Richards A Karlsson R Leonenko G Jones SE Jones HJ. Comparison of genetic liability for sleep traits among individuals with bipolar disorder I or II and control participants. JAMA Psychiatry. (2019) 77:303–10. 10.1001/jamapsychiatry.2019.4079 31751445 PMC6902167

[11] Blanken TF Benjamins JS Borsboom D Vermunt JK Paquola C Ramautar J. Insomnia disorder subtypes derived from life history and traits of affect and personality. Lancet Psychiatry. (2019) 6:151–63. 10.1016/S2215-0366(18)30464-4 30630691

[12] Bondar J Caye A Chekroud AM Kieling C. Symptom clusters in adolescent depression and differential response to treatment: a secondary analysis of the treatment for adolescents with depression study randomised trial. Lancet Psychiatry. (2020) 7:337–43. 10.1016/S2215-0366(20)30060-2 32199509

[13] United States Census Bureau. Source 2019 Population Estimates. (2020). Available online at: https://data.census.gov/cedsci/all?q=Population%20estimates/ (accessed November 2, 2020).

[14] Passarella S Duong MT. Diagnosis and treatment of insomnia. Am J Health Syst Pharm. (2008) 65:927–34. 10.2146/ajhp060640 18463341

[15] Roehrs TA Randall S Harris E Maan R Roth T. MSLT in primary insomnia: stability and relation to nocturnal sleep. Sleep. (2011) 34:1657–52. 10.5665/sleep.1426 22131601 PMC3208841

[16] Bjorøy I Jørgensen VA Pallesen S Bjorvatn B. The prevalence of insomnia subtypes in relation to demographic characteristics, anxiety, depression, alcohol consumption and use of hypnotics. Front Psychol. (2020) 11:527. 10.3389/fpsyg.2020.00527 32265811 PMC7105746

[17] Hohagen F Käppler C Schramm E Riemann D Weyerer S Berger M. Sleep onset insomnia, sleep maintaining insomnia and insomnia with early morning awakening–temporal stability of subtypes in a longitudinal study on general practice attenders. Sleep. (1994) 17:551–4. 7809569

[18] Jansen PR Watanabe K Stringer S Skene N Bryois J Hammerschlag AR. Genome-wide analysis of insomnia in 1,331,010 individuals identifies new risk loci and functional pathways. Nat Genet. (2019) 51:394–403. 10.1038/s41588-018-0333-3 30804565

[19] Stein MB McCarthy MJ Chen CY. Genome-wide analysis of insomnia disorder. Mol Psychiatry. (2018) 23:2238–50. 10.1038/s41380-018-0033-5 29520036 PMC6129221

[20] Barclay NL Gehrman PR Gregory AM Eaves LJ Silberg JL. The heritability of insomnia progression during childhood/adolescence: results from a longitudinal twin study. Sleep. (2015) 38:109–18. 10.5665/sleep.4334 25325458 PMC4262942

[21] Beaulieu-Bonneau S LeBlanc M Mérette C Dauvilliers Y Morin CM. Family history of insomnia in a population-based sample. Sleep. (2007) 30:1739– 45. 10.1093/sleep/30.12.1739 18246983 PMC2276141

[22] Spielman AJ Caruso LS Glovinsky PB. A behavioral-perspective on insomnia treatment. Psychiatr Clin North Am. (1987) 10:541–53. 10.1016/S0193-953X(18)30532-X 3332317

[23] Perlis ML Corbitt CB Kloss JD. Insomnia research: 3Ps and beyond. Sleep Med Rev. (2014) 18:191–93. 10.1016/j.smrv.2014.01.003 24685396

[24] Halonen JI Lallukka T Pentti J Stenholm S Rod NH Virtanen M. Change in job strain as a predictor of change in insomnia symptoms: analyzing observational data as a non-randomized pseudo-trial. Sleep. (2017) 40:zsw007. 10.1093/sleep/zsw007 28364463 PMC5806551

[25] Solaiman SS Agrawal R. Non-24-hour sleep-wake circadian rhythm disorder in a sighted male with normal functioning. J Clin Sleep Med. (2018) 14:483–4. 10.5664/jcsm.7008 29458698 PMC5837852

[26] Green MJ Espie CA Hunt K Benzeval M. The longitudinal course of insomnia symptoms: inequalities by sex and occupational class among two different age cohorts followed for 20 years in the west of Scotland. Sleep. (2012) 35:815–23. 10.5665/sleep.1882 22654201 PMC3353044

[27] Patel NP Grandner MA Xie D Branas CC Gooneratne N. “Sleep disparity” in the population: poor sleep quality is strongly associated with poverty and ethnicity. BMC Public Health. (2010) 10:475. 10.1186/1471-2458-10-475 20701789 PMC2927542

[28] Arber S Bote M Meadows R. Gender and socio-economic patterning of self-reported sleep problems in Britain. Soc Sci Med. (2009) 68:281–9. 10.1016/j.socscimed.2008.10.016 19026480

[29] Wu MP Lin HJ Weng SF Ho CH Wang JJ Hsu YW. Insomnia subtypes and the subsequent risks of stroke: report from a nationally representative cohort. Stroke. (2014) 45:1349–54. 10.1161/STROKEAHA.113.003675 24699057

[30] Hublin C Sallinen M. Behaviorally induced insufficient sleep. Sleep Med Clin. (2012) 7:313–23. 10.1016/j.jsmc.2012.03.008

[31] Williams AB Dzierzewski JM Griffin SC Lind MJ Dick D Rybarczyk BD. Insomnia disorder and behaviorally induced insufficient sleep syndrome: prevalence and relationship to depression in college students. Behav Sleep Med. (2020) 18:275–86. 10.1080/15402002.2019.1578772 30789063 PMC6814500

[32] Benjamins JS Migliorati F Dekker K Wassing R Moens S Blanken TF. Insomnia heterogeneity: characteristics to consider for data-driven multivariate subtyping. Sleep Med Rev. (2017) 36:71–81. 10.1016/j.smrv.2016.10.005 29066053

[33] Miller CB Bartlett DJ Mullins AE Dodds KL Gordon CJ Kyle SD. Clusters of insomnia disorder: an exploratory cluster analysis of objective sleep parameters reveals differences in neurocognitive functioning, quantitative EEG, and heart rate variability. Sleep. (2016) 39:1993–2004. 10.5665/sleep.6230 27568796 PMC5070753

[34] Laharnar N Grote L Zou D Hedner J Sommermeyer D Straßenburger C. Overnight pulse wave analysis to assess autonomic changes during sleep in insomnia patients and healthy sleepers. PLoS ONE. (2020) 15:e0232589. 10.1371/journal.pone.0232589 32379833 PMC7205215

[35] Grandner MA. Sleep duration across the lifespan: implications for health. Sleep Med Rev. (2012) 16:199–201. 10.1016/j.smrv.2012.02.001 22406305 PMC3726209

[36] Espie CA Kyle SD Hames P Gardani M Fleming L Cape J. The sleep condition indicator: a clinical screening tool to evaluate insomnia disorder. BMJ Open. (2014) 4:e004183. 10.1136/bmjopen-2013-004183 24643168 PMC3964344

[37] Regestein QR Bambrosia J Hallett M Murawski B Paine M. Daytime alertness in patients with insomnia. Am J Psychiatry. (1993) 150:1529–34. 10.1176/ajp.150.10.1529 8379559

[38] Khassawneh BY Bathgate CJ Tsai SC Edinger JD. Neurocognitive performance in insomnia disorder: the impact of hyperarousal and short sleep duration. J Sleep Res. (2018) 27:e12747. 10.1111/jsr.12747 30069961

[39] Li Y Vgontzas AN Fernandez-Mendoza J Bixler EO Sun Y Zhou J. Insomnia with physiological hyperarousal is associated with hypertension. Hypertension. (2015) 65:644–50. 10.1161/HYPERTENSIONAHA.114.04604 25624338

[40] Drake C Richardson G Roehrs T Scofield H Roth T. Vulnerability to stress-related sleep disturbance and hyperarousal. Sleep. (2004) 27:285–91. 10.1093/sleep/27.2.285 15124724

[41] Espie CA Kyle SD Hames P Cyhlarova E Benzeval M. The daytime impact of DSM-5 insomnia disorder: comparative analysis of insomnia subtypes from the Great British Sleep Survey. J Clin Psychiatry. (2012) 73:e1478–84. 10.4088/JCP.12m07954 23290331

[42] Baglioni C Altena E Bjorvatn B Blom K Bothelius K Devoto A. The European academy for cognitive behavioural therapy for insomnia: an initiative of the European insomnia network to promote implementation and dissemination of treatment. J Sleep Res. (2020) 29:e12967. 10.1111/jsr.12967 31856367

[43] Suraev A Grunstein RR Marshall NS D'Rozario AL Gordon CJ Bartlett DJ. Cannabidiol (CBD) and Δ(9)-tetrahydrocannabinol (THC) for chronic insomnia disorder ('CANSLEEP' trial): protocol for a randomised, placebo-controlled, double-blinded, proof-of-concept trial. BMJ Open. (2020) 10:e034421. 10.1136/bmjopen-2019-034421 PMC7239553 32430450

[44] Merikanto I Kronholm E Peltonen M Laatikainen T Lahti T Partonen T. Relation of chronotype to sleep complaints in the general Finnish population. Chronobiol Int. (2012) 29:311–7. 10.3109/07420528.2012.655870 22390244

[45] Bjorvatn B Jernelöv S Pallesen S. Insomnia – A heterogenic disorder often comorbid with psychological and somatic disorders and diseases: a narrative review with focus on diagnostic and treatment challenges. Front Psychol. (2021) 12:639198. 10.3389/fpsyg.2021.639198 33643170 PMC7904898

[46] Mikoteit T Brand S Eckert A Holsboer-Trachsler E Beck J. Brain-derived neurotrophic factor is a biomarker for subjective insomnia but not objectively assessable poor sleep continuity. J Psychiatr Res. (2019) 110:103–9. 10.1016/j.jpsychires.2018.12.020 30616157

[47] Neumann N Lotze M Domin M. Sex-specific association of poor sleep quality with gray matter volume. Sleep. (2020) : zsaa035. 10.1093/sleep/zsaa035 PMC7487870 32140718

[48] Collen J York CM. Wrist wearables: more questions than answers? J Clin Sleep Med. (2019) 15:1077–78. 10.5664/jcsm.7858 31482827 PMC6707053

[49] Buysse DJ Reynolds CF Monk TH Berman SR Kupfer DJ. The pittsburgh sleep quality index – a new instrument for psychiatric practice and research. Psychiat Res. (1989) 28:193–213. 10.1016/0165-1781(89)90047-4 2748771

[50] Espie CA Inglis SJ Harvey L Tessier S. Insomniacs' attributions: psychometric properties of the dysfunctional beliefs and attitudes about sleep scale and the sleep disturbance questionnaire. J Psychosom Res. (2009) 48:141–8. 10.1016/s0022-3999(99)00090-2 10719130

[51] Palagini L Cellini N Mauri M Mazzei I Simprage S dell'Osso L. Multiple phenotypes of resting-state cognition are altered in insomnia disorder. Sleep Health. (2016) 2:239–45. 10.1016/j.sleh.2016.05.003 29073428

[52] Ree MJ Harvey AG. Investigating safety behaviours in insomnia: the development of the sleep-related behaviours questionnaire (SRBQ). Behav Change. (2004) 21:26–36. 10.1375/bech.21.1.26.35971

[53] Bell C McLeod LD Nelson LM Fehnel SE Zografos LJ Bowers B. Development and psychometric evaluation of a new patient-reported outcome instrument measuring the functional impact of insomnia. Qual Life Res. (2011) 20:1457–68. 10.1007/s11136-011-9885-8 21505882

[54] Tarrasch R1 Laudon M Zisapel N. Cross-cultural validation of the Leeds Sleep Evaluation Questionnaire (LSEQ) in insomnia patients. Hum Psychopharmacol. (2003) 18:603–10. 10.1002/hup.534 14696019

[55] Broomfield NM Espie CA. Towards a valid, reliable measure of sleep effort. J Sleep Res. (2005) 14:401–7. 10.1111/j.1365-2869.2005.00481.x 16364141

[56] Luik AI Machado PF Siriwardena N Espie CA. Screening for insomnia in primary care: using a two-item version of the Sleep Condition Indicator. Br J Gen Pract. (2019) 69:79–80. 10.3399/bjgp19X701045 30705006 PMC6355290

[57] Kalmbach DA Cheng P O'Brien LM Swanson LM Sangha R Sen S. A randomized controlled trial of digital cognitive behavioral therapy for insomnia in pregnant women. Sleep Med. (2020) 72:82–92. 10.1016/j.sleep.2020.03.016 32559716 PMC8210706

